# Capillary leak syndrome following COVID-19 vaccination: Data from the European pharmacovigilance database Eudravigilance

**DOI:** 10.3389/fimmu.2022.956825

**Published:** 2022-09-13

**Authors:** Rosanna Ruggiero, Nunzia Balzano, Raffaella Di Napoli, Annamaria Mascolo, Pasquale Maria Berrino, Concetta Rafaniello, Liberata Sportiello, Francesco Rossi, Annalisa Capuano

**Affiliations:** ^1^ Campania Regional Centre for Pharmacovigilance and Pharmacoepidemiology, University of Campania “L. Vanvitelli”, Naples, Italy; ^2^ Department of Experimental Medicine – Section of Pharmacology “L. Donatelli”, University of Campania “L. Vanvitelli”, Naples, Italy; ^3^ Department of Specialized Medicine, Diagnostic and Experimental, University of Bologna “Alma Mater Studiorum”, Bologna, Italy

**Keywords:** COVID-19 vaccines, capillary leak syndrome, safety, pharmacovigilance, Eudravigilance, plausibility, AEFI, hypercytokinemia

## Abstract

Capillary leak syndrome (CLS) emerged as new adverse event after immunization (AEFI) associated to COVID-19 vaccination. CLS is a rare condition characterized by increased capillary permeability, resulting in hypoalbuminemia, hypotension, and edema mainly in the upper and lower limbs. Our pharmacovigilance study aims to evaluate the CLS onset following receipt of COVID-19 mRNA vaccines (mRNA-1273 and BNT162b2) compared to viral vector vaccines (Ad26.COV2-S and ChAdOx1-SARS-COV-2). We carried a cross-sectional study using all Individual Case Safety Reports (ICSRs) reporting a COVID-19 vaccine as suspected drug and CLS as AEFI, which were collected in the pharmacovigilance database EudraVigilance from January 1st, 2021, to January 14th, 2022. We applied the Reporting Odds Ratio (ROR) 95% CI for the disproportionality analysis. During our study period, CLS was described as AEFI in 84 out of 1,357,962 ICRs reporting a vaccine COVID-19 as suspected drug and collected in the EV database. Overall, the ICSR reported by CLS were mainly related to the viral vector COVID-19(ChAdOx1-SARS-COV-2 = 36; Ad26.COV2-S = 9). The mRNA COVID-19 vaccines were reported in 39 ICSRs (BNT162b2 =33; mRNA-1273 =6). Majority of ICSRs were reported by healthcare professionals (71.4%). Majority of the patients were adult (58.3%) and the female gender accounted in more than 65% of ICSRs referred both to classes vaccines. In particular, women were more represented in ICSRs referred to mRNA-1273 (83.3%) and to ChAdOx1-SARS-COV-2 (72.2%). The CLS outcome was more frequently favorable in mRNA ICSRs (33,3%) than the viral vector ones (13.3%). Among the ICSRs reporting CLS with unfavorable outcome, we found also 9 fatal cases (BNT162b2 = 1; ChAdOx1-SARS-COV-2 = 4; Ad26.COV2-S = 4). From disproportionality analysis emerged a lower CLS reporting probability after vaccination with mRNA vaccines compared to viral vector-based ones (ROR 0.5, 95% CI 0.3–0.7; p <0.001).Our findings, even if subject to the limitations of spontaneous reporting systems, suggest a small but statistically significant safety concern for CLS following receipt of COVID-19 viral vector vaccines, in particular with Ad26.COV2-S. Cytokine-release following T-cell activation could be involved in CLS occurrence, but a precise mechanism has been not yet identified. COVID-19 vaccines remain attentive as possible triggers of CLS.

## Introduction

Since the outbreak of a pneumonia of unknown etiology, the attention of scientific and civil community has been focusing on a new coronavirus, Severe Acute Respiratory Syndrome Coronavirus 2 (SARS-CoV-2) that causes Coronavirus Disease 2019 (COVID-19) (1). COVID-19 results in various symptoms, including the classic ones of upper respiratory tract infections, and also myalgia, hemoptysis, fatigue. In a substantial percentage of patients, it can evolve into a serious, potentially fatal, pneumonia with wheezing, chest tightness, shortness of breath and severe chest signs (2). This global health emergency determined the need of health interventions to control and prevent COVID-19 (3–5). So, the worldwide scientific community collaborated to find effective vaccines for stemming the pandemic (6). Considering the special circumstances, regulatory agencies have resorted to exceptional strategies and procedures to accelerate the processes of efficacy, quality and safety evaluation for new anti-COVID19 drugs and vaccines (4), such as the rolling review used by the European Medicines Agency (EMA) (7). So, after only one year of pandemic, the first vaccine against COVID-19, BNT162b2, received a conditional marketing authorization by EMA (8). Currently, in the European Union five vaccines based on different platforms have been authorized: two mRNA vaccines (BNT162b2 and mRNA-1273 ), two viral vector vaccines (COVID-19 Vaccine Ad26.COV2-S and ChAdOx1-SARS-COV-2 ) and an adjuvanted vaccine containing a SARS-CoV-2 recombinant spike protein (9). Receiving a conditional approval, these vaccines are under additional monitoring. In light of this, reporting, collection and analysis of the adverse events after immunization (AEFIs) became even more important to extrapolate safety information (10). A strong pharmacovigilance system, including routine and additional pharmacovigilance activities, is require to monitor the safety and the effectiveness of COVID-19 vaccines through data coming from the real world (11). Thanks to the spontaneous reporting, less common AEFIs, such as vaccine-associated capillary leak syndrome (CLS), emerged (12). Some reports described this rare syndrome occurring mainly in people who received ChAdOx1-SARS-COV-2 or Ad26.COV2-S COVID-19 vaccine (from now on named as Ad26.COV2-S ) (13–15). CLS is characterized by an increase of capillary permeability, resulting in a condition of hypoalbuminemia, hypotension and edema. This condition is rare, but serious and potentially fatal (16). The increasing in spontaneous reports of CLS after vaccinations, induced EMA to advise against use of Ad26.COV2-S and ChAdOx1-SARS-COV-2 in subjects who previously experienced this condition (17, 18). Moreover, some case reports describing CLS following SARS-CoV-2 infection and after COVID-19 vaccination with mRNA vaccines are described in literature (19, 20). Considering the clinical significance of this new side effect, our study aims to assess the occurrence of CLS following the immunization with COVID-19 mRNA vaccines compared to viral vector vaccines by analyzing the data collected in the European spontaneous reporting system database, Eudravigilance (EV) (21).

## Materials and methods

### Data source

For this cross-sectional study, we selected all ICSRs reporting mRNA-1273, BNT162b2, Ad26.COV2-S or ChAdOx1-SARS-COV-2 as a suspected drug and CLS as AEFI, collected in EV from January 1^st^, 2021 to January 14^th^, 2022. The adjuvanted vaccine containing a SARS-CoV-2 recombinant spike protein has been excluded since as of January 14^th^, 2022 its administration was not yet started in EU. The EV is the European pharmacovigilance database, handled by the EMA, which collects all ICSRs of suspected adverse drug reactions (ADRs) and AEFI reported to an EU national competent authority or a marketing authorization holder. For transparency, some EV data are publicly available through the EMA website (www.adrreports.eu). Analysis of large pharmacovigilance databases, such as EV, allows the extrapolation of important safety information coming from the real world context (21).

### Descriptive analyses

Data were provided for each authorized COVID-19 vaccine, categorized based on their used platforms (viral vector or mRNA). We performed a systematic analysis of dataset providing information’s about ICSR collection (monthly trend, reporter type and the country for regulatory purposes), patients characteristics (age group and gender), adverse events (duration, outcome, seriousness and overlapping with other AEFIs) and treatments (including concomitant drugs). All AEFIs were categorized based on the seriousness criteria in accordance with the International Council on Harmonization E2D guidelines. The overlapped AEFIs, reported in addition to CLS, were categorized according the Medical Dictionary for Regulatory Activities (MedDRA ) in the reference System Organ Class (SOC) and High-Level Group Term (HLGT). The concomitant drugs were classified according to the second level of the Anatomical Therapeutic Chemical (ATC) classification system of World Health Organization.

### Disproportionality analysis

To compare the frequency of CLS reporting for each COVID-19 vaccine, we performed the Reporting Odds Ratio (ROR) 95% CI. ROR was computed within the same vaccine class (classified in mRNA and viral vector vaccines) and comparing the two COVID-19 vaccines classes. The RORs were computed on ICSRs numbers since these are publicly available on the EMA website. Forest plots were performed for both comparisons using R (Version 4.1.2 (2021-11-01); R Development Core Team).

## Results

### Descriptive analysis

**Figure 1:**
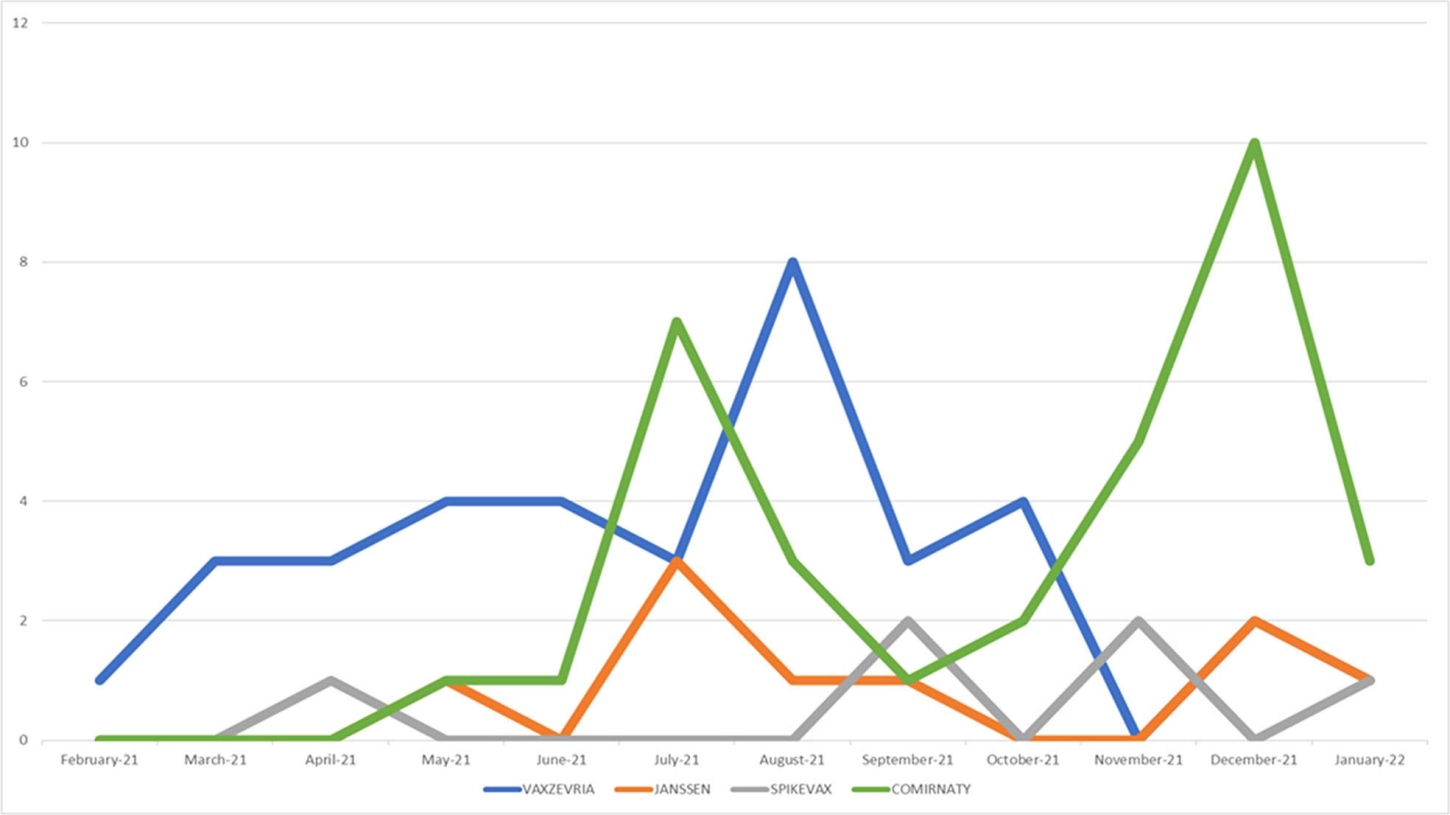
Trends of spontaneous reporting of CLS from EudraVigilnce database between January 1_st_, 2021 and January 14_th_, 2022.

During our study period, CLS was described as AEFI in 84 out of 1,357,962 ICSRs reporting a vaccine COVID-19 as suspected drug and collected in the EV database. In 2021 the reporting monthly trend showed a volatile pattern with highest peaks between July and November, mainly referred to mRNA-1273 and Ad26.COV2-S vaccines (**Figure 1**). The first CLS cases were reported starting from February 2021 and they were referred to ChAdOx1-SARS-COV-2, for which a more sustained reporting trend emerged over time.

Overall, the CLS-reporting ICSRs were mainly referred to COVID-19 viral vector vaccines, reported as suspected drug in overall 45 ICSRs (ChAdOx1-SARS-COV-2, N=36; Ad26.COV2-S, N=9), while 39 ICSRs pertained to COVID-19 mRNA vaccines (BNT162b2, N=33; mRNA-1273, N=6). Characteristics of all ICSR reporting CLS as AEFI with COVID-19 vaccines were presented in **Table 1**. We found only one case of CLS referred to heterologous prime-boost vaccination scheme. In this case, the patient was an adult female vaccinated with Vaxzervria (prime) and BNT162b2 (booster), who also manifested necrosis skin, finger amputation, blisters, vasculitis in addition to CLS. At the moment of reporting, the case was not resolved resulting in patient disability.

**Table 1:** Demographic characteristics of Individual Case Safety Reports (ICSRs) involving COVID19 vaccines reported in the EudraVigilance spontaneous reporting system from 1st January 2021 to 14^th^ January 2022.

Variable	Level	All mRNA Vaccine (n=39)	All Viral Vector Vaccine (n=45)	BNT162b2 (n=33)	mRNA-1273 (n=6)	ChAdOx1-SARS-COV-2 (n=36)	Ad26.COV2-S (n=9)
***Age***	*< 18 years (%)*	0	0	0	0	0	0
	*18-64 years (%)*	27 (69,2%)	22 (48.9%)	21 (63.7%)	6 (100%)	16 (44.4%)	6 (66.7%)
	*> 65 years (%)*	11 (28.2%)	18 (40.0%)	11 (33.3%)	0	15 (41.7%)	3 (33.3%)
	*Missing (%)*	1 (2.6%)	5 (11.1%)	1 (3.0%)	0	5 (13.9%)	0
***Gender***	*F (%)*	27 (69.2%)	30 (66.7%)	22 (66.7%)	5 (83.3%)	26 (72.2%)	4 (44.4%)
	*M (%)*	12 (30.8%)	13 (28.9%)	11 (33.3%)	1 (16.7%)	8 (22.2%)	5 (55.6%)
	*Missing (%)*	0	2 (4.4%)	0	0	2 (5.6%)	0
***Seriousness of Capillary leak syndrome***	*Serious (%) of which: Other Medically Important Condition Results in Death Caused/Prolunged Hospitalisation Life Threatening Disabling*	37 (94.9%)13 (33.3%)1 (2.6%)9 (23.1%)13 (33.3%)1 (2.6%)	39 (86.7%)13 (39.3%)10 (3.0%)5 (21.2%)10 (30.0%)1 (6.0%)	31 (93.9%)13 (41.9%)1 (3.2%)7 (22.6%)10 (32,3%)0	6 (100%)002 (33.3%)3 (50.0%)1 (16.7%)	30 (83.3%)12 (40.0%)4 (13.3%)4 (13.3%)9 (30.0%)1 (3.3%)	9 (100%)1 (11.1%)6 (66.7%)1 (11.1%)1 (11.1%)0
	*Not available (%)*	2 (5.1%)	6 (13.3%)	2 (6.1%)	0	6 (16.7%)	0
***Outcome of Capillary leak syndrome***	*Fatal (%)*	1 (2,6%)	8 (17.8%)	1 (3,0%)	0	4 (11.1%)	4 (44.4%)
	*Not Recovered/Not Resolved (%)*	7 (17,9%)	8 (17.8%)	6 (18,2%)	1 (16,7%)	7 (19.4%)	1 (11.1%)
	*Recovered/Resolved (%)*	13 (33,3%)	6 (13.3%)	8 (24,2%)	5 (83,3%)	6 (16.7%)	0
	*Recovered/Resolved With Sequelae (%)*	1 (2,6%)	0	1 (3,0%)	0	0	0
	*Recovering/Resolving (%)*	9 (23,1%)	10 (22.2%)	9 (27,3%)	0	9 (25.0%)	1 (11.1%)
	*Unknown (%)*	8 (20,5%)	13 (28.9%)	8 (24,3%)	0	10 (27.8%)	3 (33.3%)
***Capillary leak syndrome Duration***	*Mean (IQR)*	13.7 d (8.0-19)	9.5 d (4.5-13.5)	11.5 d (6.5-17)	6 d (3.3-7.3)	8 d (5.5-10.5)	25 d
***Primary Source***	*Healthcare Professional (%)*	30 (76.9%)	30 (66.7%)	24 (72.7%)	6 (100%)	21 (58.3%)	9 (100%)
	*Non-Healthcare Professional (%)*	9 (30.1%)	15 (33.3%)	9 (27.3%)	0	15 (41.7%)	0
***Primary Source Country for Regulatory Purposes***	*European Economic Area (%)*	26 (66.7%)	26 (57.8%)	23 (69.7%)	3 (50.0%)	20 (55.6%)	6 (66.7%)
	*Non-European Economic Area (%)*	13 (33.3%)	19 (42.2%)	10 (30.3%)	3 (50.0%)	16 (44.4%)	3 (33.3%)
***Concomitant drugs***	*1 (%)*	3 (7.7%)	5 (11.1%)	2 (6.1%)	1 (16.7%)	3 (8.3%)	2 (22.2%)
	*2 (%)*	3 (7.7%)	4 (8.9%)	2 (6.1%)	1 (16.7%)	4 (11.2%)	0
	*3 (%)*	1 (2.6%)	0	0	1 (16.7%)	0	0
	*4 (%)*	2 (5.1%)	1 (2.2%)	1 (3.0%)	1 (16.7%)	1 (2.8%)	0
	≥ 5 *(%)*	3 (7.7%)	5 (11.1%)	1 (3.0%)	2 (33.2%)	3 (8.3%)	2 (22.2%)
	*Missing (%)*	27 (69.2%)	30 (66.7%)	27 (81.8%)	0	25 (69.4%)	5 (55.6%)

Overall, the majority of the patients were adult (N=49; 58.3%). Female gender accounted in more than 65% of ICSRs referred both to mRNA vaccines and viral vector vaccines. In particular, women were more represented in ICSRs referred to mRNA-1273 (83.3%) and to ChAdOx1-SARS-COV-2 (72.2%). Majority of ICSRs were reported by healthcare professionals (N=60; 71.4%). Non-healthcare professionals represented the primary source in the 41.7% of ChAdOx1-SARS-COV-2 -related ICSRs. In terms of the primary source country for regulatory purposes, the European Economic Area was the most representative one for BNT162b2 (N=23; 69.7%), ChAdOx1-SARS-COV-2 (N=20; 55.6%) and Ad26.COV2-S (N=6%; 66.7%) ICSRs, while those pertained to mRNA-1273 were equally distributed between European and Non-European Economic Area (50%). Although the seriousness was unspecified in 8 ICSRs, CLS resulted a serious AEFI in 90.5% of ICSRs (N=76), representing a life threatening for the patient in 23 cases (27.4%), requiring or prolonging hospitalization in 14 cases (16,7%) and causing patient’s death in 11 cases (13.1%). In particular, these cases were mainly referred viral vector vaccines, specifically to Ad26.COV2-S (N=6), followed by ChAdOx1-SARS-COV-2 (N=4). The remaining case was referred to BNT162b2. The mean CLS duration, reported only in 14 out of 84 ICSRs, was 10.4 days (IQR: 4.3–13,8). In 45.2% of ICSRs (N=38), the CLS outcome was favorable, resulting in a complete resolution or improvement (N=19; both). In particular, 83.3% of mRNA-1273 -related CLS (N=5) was completely resolved. On the other hand, in 29.8% of ICSRs (N=25) the CLS outcome was unfavorable resulting as resolved with sequelae, unchanged or fatal in 1.2% (N=1), in 17.9% (N=15) or in 10.7% (N=9) of ICSR, respectively. The reported CLS with fatal *exitus* pertained mainly to ChAdOx1-SARS-COV-2 (N=4) and Ad26.COV2-S (N=4), while only one BNT162b2 -related fatal case emerged (**Table 1**). CLS exacerbation, lasted 98h and completely resolved after treatment was reported in only one ICSR, referred to a female patient vaccinated with mRNA-1273 .

**Figure 2:**
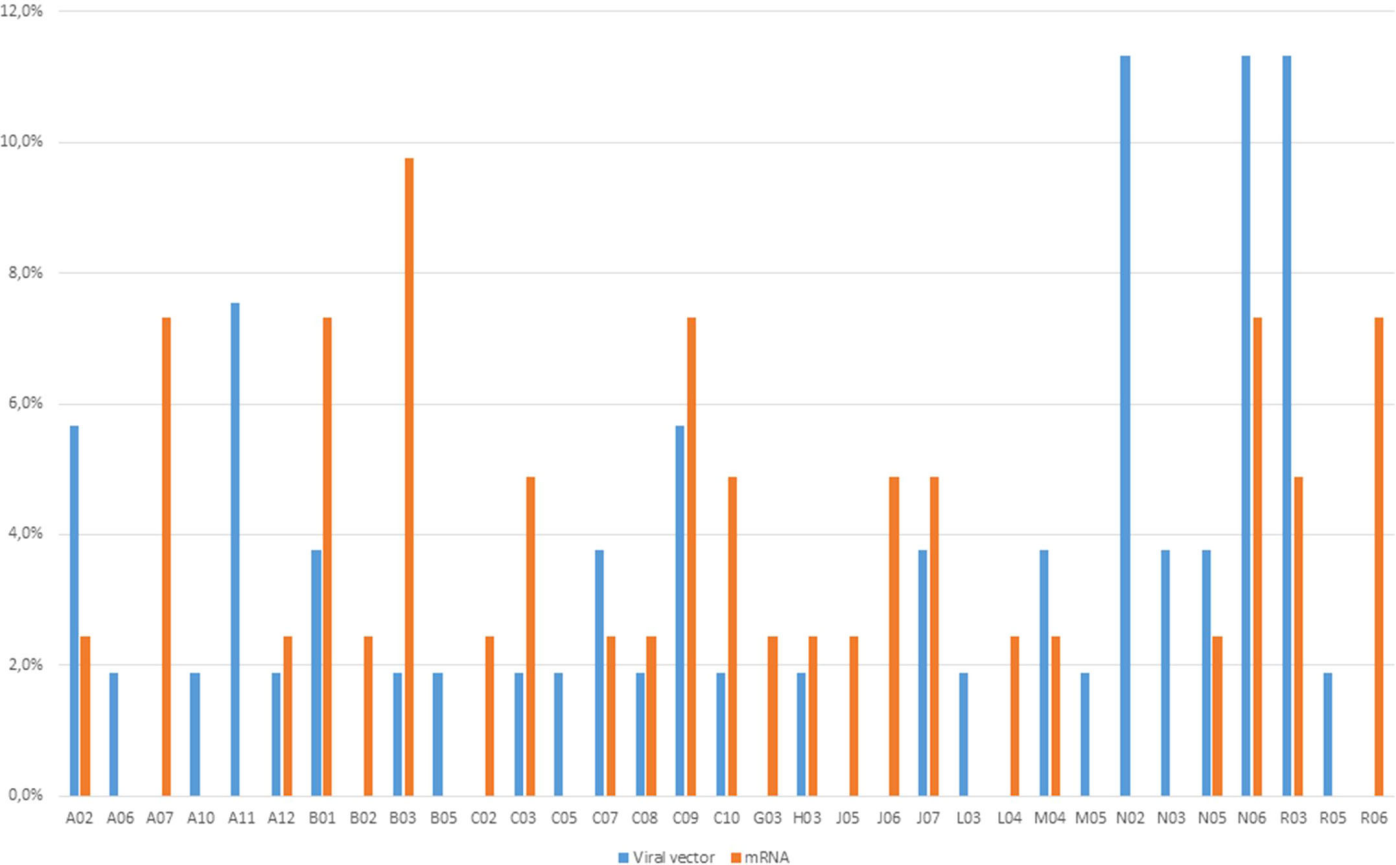
Distribution of other concomitant drugs reported in the ICSR related to mRNA- and viral vector-based COVID-19 vaccines.

In the majority of ICSRs (N=57; 67.9%) the concomitant drugs were missing, but in 27 ICSRs it was reported at least a concomitant drug. More than 5 concomitant drugs were reported in 8 ICSRs, that were mainly referred to ChAdOx1-SARS-COV-2 (N=3), following by Ad26.COV2-S and mRNA-1273 (N=2, both) and BNT162b2 (N=1). Generally, psychoanaleptics (N=9) were the most frequently reported concomitant agents, followed by drugs for obstructive airway diseases (N=8), agents acting on the renin-angiotensin system and analgesics (N=6, both). Distribution of other suspected or concomitant drugs reported in the ICSR related to mRNA and viral vector vaccines is reported in **Figure 2**.

The 82.1% of ICSRs related to COVID-19 mRNA vaccines (N=26, 78.8% for BNT162b2; N=6, 100% for mRNA-1273) reported additional AEFIs overlapping with CLS, accounting overall 160 and 34 other adverse events for BNT162b2 and mRNA-1273, respectively. For both mRNA vaccines, the overlapping AEFI were mainly general disorders and administration site conditions, including body temperature conditions. These ones were followed by events belonging to “Respiratory, thoracic and mediastinal disorders” SOC in BNT162b2 -related cases (11.8%), and “Vascular disorders” SOC for mRNA-1273 (14.7%) (**Table 2**). Considering the viral vector vaccines, other AEFIs overlapping to CLS were reported in 80% of ICSRs. As with mRNA vaccines, the majority of other overlapped AEFI belonged to “General disorders and administration site conditions” SOC, followed by “Vascular disorders” one, including events referred to the “Decreased and nonspecific blood pressure disorders and shock” and “Embolism and thrombosis” HGLTs. Moreover, in ChAdOx1-SARS-COV-2 -related ICSR, neurologic disorders overlapped with CLS. In particular, the events belonged to “Peripheral neuropathies”, “Headaches”, “Movement disorders (incl parkinsonism)” and “Central nervous system vascular disorders” HLGTs (**Table 2**).

**Table 2:** Distribution of adverse events overlapping with CLS and reported in all COVID-19 Vaccines ICSRs collected in the EudraVigilance spontaneous reporting system from January 1_st_, 2021 to January 14_th_, 2022, categorized by MedDRA System Organ Class, and High-Level Group Terms (HLGT) within each System Organ Class (SOC).

Adverse events by MedDRA SOC and HLGT	TOT	BNT162b2 Adverse events	mRNA-1273 Adverse events	ChAdOx1-SARS-COV-2 Adverse events	COVID-19 vaccine Ad26.COV2-S
		N=160 (%)	N=34 (%)	N=185 (%)	Adverse events
					N=43 (%)
***Blood and lymphatic system disorders***	**10 (2.4)**	**1 (0.6)**	**1 (3.0)**	**5 (2.7)**	**3 (1.6)**
Anaemias nonhaemolytic and marrow depression	1 (10.0)	1 (100)	–	–	–
Coagulopathies and bleeding diatheses (excl thrombocytopenic)	2 (20.0)	–	–	1 (20.0)	1 (33.3)
Haematological disorders NECSpleen, lymphatic and reticuloendothelial system disordersSpleen, lymphatic and reticuloendothelial system disorders	1 (10.0)	–	1 (100)	–	–
Haemolyses and related conditions	1 (10.0)	–	–	–	1 (33.3)
Platelet disorders	2 (20.0)	–	–	1 (20.0)	1 (33.3)
Spleen, lymphatic and reticuloendothelial system disorders	2 (20.0)	–	–	2 (40.0)	–
White blood cell disorders	1 (10.0)	–	–	1 (20.0)	–
***Cardiac disorders***	**13 (3.1)**	**9 (5.6)**	**1 (3.0)**	**3 (1.6)**	**-**
Cardiac arrhythmias	5 (38.5)	4 (44.5)	–	1 (33.3)	–
Cardiac disorders, signs and symptoms NEC	1 (7.7)	1 (11.1)	–	–	–
Heart failures	2 (15.4)	1 (11.1)	–	1 (33.3)	–
Myocardial disorders	2 (15.4)	1(11.1)	–	1 (33.3)	–
Pericardial disorders	3(23.1)	2 (22.2)	1 (100)	–	–
***Ear and labyrinth disorders***	**1 (0.2)**	**1 (0.6)**	**-**	–	–
Hearing disorders	1 (100)	1 (100)	–	–	–
***Endocrine disorders***					** **
*** ***	**1 (0.2)**	–	–	–	**1 (0.5)**
Thyroid gland disorders	1 (100)	–	–	–	1 (100)
***Eye disorders***	**8 (1.9)**	**4 (2.5)**	**1 (3.0)**	**2 (1.1)**	**1 (0.5)**
Eye disorders NEC	2 (25.0)	1 (25.0)	–	–	1 (100)
Inner ear and VIIIth cranial nerve disorders	1 (12.5)	–	1 (100)	–	–
Ocular infections, irritations and inflammations	1 (12.5)	–	–	1 (50.0)	–
Vision disorders	4 (50.0)	3 (75.0)	–	1 (50.0)	–
***Gastrointestinal disorders***	**28 (6.6)**	**12 (7.5)**	**4 (11.7)**	**9 (4.9)**	** **
*** ***	** **	** **	** **	** **	**3 (1.6)**
Gastrointestinal inflammatory conditions	3 (10.7)	1 (8.3)	–	1 (11.1)	1 (33.3)
Gastrointestinal motility and defaecation conditions	1 (3.6)	1 (8.3)	–	–	–
Gastrointestinal signs and symptoms	22 (78.6)	9 (75.1)	4 (100)	7 (77.8)	2 (66.7)
Oral soft tissue conditions	2 (7.1)	1(8.3)	–	1 (11.1)	–
***General disorders and administration site conditions***	**96 (22.7)**	**36 (22.5)**	**11 (32.3)**	**35 (18.9)**	**14 (7.6)**
Administration site reactions	4 (4.2)	3 (8.3)	–	1 (2.9)	–
Body temperature conditions	10 (10.4)	3 (8.3)	1 (9.1)	4 (11.4)	2 (14.3)
Fatal outcomes	2 (2.1)	–	–	1 (2.9)	1 (7.1)
General system disorders NEC	77 (80.2)	28 (77.8)	10 (90.9)	29 (82.8)	10 (71.5)
Therapeutic and nontherapeutic effects (excl toxicity)	1 (1.0)	1 (2.8)	–	–	–
Tissue disorders NEC	2 (2.1)	1 (2.8)	–	–	1 (7.1)
***Immune disorders NEC***	** **	** **			
*** ***	**4 (0.9)**	**3 (1.8)**	–	**1 (0.5)**	–
Immune disorders NEC	2 (50.0)	2 (66.7)	–	–	–
Allergic conditions	1 (25.0)	1 (33.3)	–	–	–
Other Medically Important Condition	1 (25.0)	–	–	1 (100)	–
***Infections and infestations***					** **
*** ***	**6 (1.4)**	**1 (0.6)**	–	**2 (1.1)**	**3 (1.6)**
Bacterial infectious disorders	1 (16.7)	–	–	1 (50.0)	–
Infections - pathogen unspecified	5 (83.3)	1 (100)	–	1 (50.0)	3 (100)
***Injury, poisoning and procedural complications***				** **	
*** ***	**18 (4.3)**	**9 (5.6)**	–	**9 (4.9)**	–
Bone and joint injuries	2 (11.1)	2 (22.2)	–	–	–
Injuries NEC	9 (50.0)	4 (44.5)	–	5 (55.6)	–
Medication errors and other product use errors and issues	4 (22.2)	2 (22.2)	–	2 (22.2)	–
Off label uses and intentional product misuses/use issues	1 (5.6)	1 (11.1)	–	–	–
Procedural related injuries and complications NEC	2 (11.1)	–	–	2 (22.2)	–
***Investigation***	**21 (5.0)**	**4 (2.5)**	**2 (5.8)**	**11 (5.9)**	**4 (2.2)**
Cardiac and vascular investigations (excl enzyme tests)	4 (19.0)	2 (50.0)	–	2 (18.2)	–
Haematology investigations (incl blood groups)	9 (42.9)	2 (50.0)	2 (100)	5 (45.4)	–
Investigations, imaging and histopathology procedures NEC	1 (4.8)	–	–	–	1 (25.0)
Metabolic, nutritional and blood gas investigations	1 (4.8)	–	–	1 (9.1)	–
Microbiology and serology investigations	1 (4.8)	–	–	–	1 (25.0)
Physical examination and organ system status topics	2 (9.5)	–	–	1 (9.1)	1 (25.0)
Protein and chemistry analyses NEC	3 (14.3)	–	–	2 (18.2)	1 (25.0)
***Metabolism and nutrition disorder***	**13 (3.1)**	**3 (1.8)**	**1 (3.0)**	**8 (4.3)**	**1 (0.5)**
Acid-base disorders	1 (7.7)	–	–	1 (12.5)	–
Electrolyte and fluid balance conditions	6 (46.2)	1 (33.3)	–	5 (62.5)	–
Protein and amino acid metabolism disorders NEC	6 (46.2)	2 (66.7)	1 (100)	2 (25.0)	1 (100)
***Musculoskeletal and connective tissue disorders***	**32 (7.6)**	**10 (6.2)**	**1 (3.0)**	**19 (10.3)**	**2 (1.1)**
Joint disorders	2 (6.3)	2 (20.0)	–	–	–
Muscle disorders	10 (31.3)	5 (50.0)	–	4 (21.1)	1 (50.0)
Musculoskeletal and connective tissue disorders NEC	19 (59.4)	3 (30.0)	1 (100)	14 (73.6)	1 (50.0)
Tendon, ligament and cartilage disorders	1 (3.1)	–	–	1 (5.3)	–
***Neoplasms benign, malignant and unspecified (incl cysts and polyps)***					** **
*** ***	**5 (1.2)**	–	–	**4 (2.2)**	**1 (0.5)**
Haematopoietic neoplasms (excl leukaemias and lymphomas)	2 (40.0)	–	–	2 (50.0)	–
Miscellaneous and site unspecified neoplasms malignant and unspecified	1 (20.0)	–	–	–	1 (100)
Plasma cell neoplasms					
	2 (40.0)	–	–	2 (50.0)	–
***Nervous system disorders***	**43 (10.2)**	**16 (10)**	**1 (3.0)**	**24 (13.0)**	**2 (1.1)**
Central nervous system vascular disorders	1 (6.3)	1 (6.2)	–	1 (8.3)	–
Headaches	2 (12.5)	2 (12.5)	–	4 (16.7)	–
Movement disorders (incl parkinsonism)	2 (12.5)	2 (12.5)	–	2 (8.3)	–
Neurological disorders NEC	10 (62.5)	10 (62.6)	1 (100)	13 (54.1)	2 (100)
Peripheral neuropathies Plasma cell neoplasms	1 (6.3)	1 (6.2)	–	4 (16.7)	–
***Psychiatric disorders***	**6 (1.4)**	**2 (1.25)**	**1 (3.0)**	**3 (1.6)**	**-**
Adjustment disorders (incl subtypes)	1 (16.7)	1 (50.0)	–	–	–
Deliria (incl confusion)	1 (16.7)	–	1 (100)	–	–
Eating disorders and disturbances	1 (16.7)	1 (50.0)	–	–	–
Somatic symptom and related disorders	1 (16.7)	–	–	1 (33.3)	–
Sleep disorders and disturbances	2 (33.3)	–	–	2 (66.7)	–
***Renal and urinary disorders***					** **
*** ***	**8 (1.9)**	**3 (1.8)**	**-**	**4 (2.2)**	**1 (0.5)**
Renal disorders (excl nephropathies)	6 (75.0)	1 (33.3)	–	4 (100)	1 (100)
Nephropathies	1 (12.5)	1(33.3)	–	–	–
Urinary tract signs and symptoms	1 (12.5)	1(33.3)	–	–	–
***Respiratory, thoracic and mediastinal disorders***	**27 (6.4)**	**19 (11.8)**	**1 (3.0)**	**6 (3.2)**	**1 (0.5)**
Bronchial disorders (excl neoplasms)	1 (3.7)	1 (5.3)	–	–	–
Lower respiratory tract disorders (excl obstruction and infection)	2 (7.4)	2 (10.5)	–	–	–
Pleural disorders	2 (7.4)	2 (10.5)	–	–	–
Respiratory disorders NEC	18 (66.7)	11 (57.9)	1 (100)	5 (83.3)	–
Respiratory tract signs and symptoms	3 (11.1)	2 (10.5)	–	1 (16.7)	1 (100)
Upper respiratory tract disorders (excl infections)	1 (3.7)	1 (5.3)	–	–	–
***Skin and subcutaneous tissue disorders***	**28 (6.6)**	**14 (8.7)**	**3 (8.2)**	**10 (5.4)**	**1 (0.5)**
Angioedema and urticaria	1 (3.6)	–	–	1 (10.0)	–
Epidermal and dermal conditions	17 (60.7)	10 (71.4)	2 (66.7)	5 (50.0)	–
Skin appendage conditions	3 (10.7)	–	1 (33.3)	1 (10.0)	1 (100)
Skin vascular abnormalities	7 (25.0)	4 (28.6)	–	3 (30.0)	–
***Social circumstances***	** **	** **			
*** ***	**1 (0.2)**	**1 (0.6)**	–	–	–
Lifestyle issues	1 (100)	1 (100)	–	–	–
***Surgical and medical procedures***	**3 (0.7)**	**-**	**1 (3.0)**	**2 (1.1)**	**-**
Bone and joint therapeutic procedures	1 (33.3)	–	1(100)	–	–
Soft tissue therapeutic procedures	1 (33.3)	–	–	1 (50.0)	–
Respiratory tract therapeutic procedures	1 (33.3)	–	–	1 (50.0)	–
***Vascular disorders***	**50 (11.8)**	**12 (7.5)**	**5 (14.7)**	**28 (15.1)**	**5 (2.7)**
Arteriosclerosis, stenosis, vascular insufficiency and necrosis	6 (12.0)	–	–	6 (21.4)	–
Decreased and nonspecific blood pressure disorders and shock	20 (40.0)	4 (33.3)	3 (60.0)	9 (32.2)	4 (80.0)
Embolism and thrombosis	7 (14.0)	2 (16.7)	1 (20.0)	3 (10.7)	1 (20.0)
Vascular disorders NEC	5 (10.0)	2 (16.7)	–	3 (10.7)	–
Vascular haemorrhagic disorders	6 (12.0)	2 (16.7)	–	4 (14.3)	–
Vascular hypertensive disorders	1 (2.0)	1 (8.3)	–	–	–
Vascular infections and inflammations	4 (8.0)	1(8.3)	1 (20.0)	2 (7.1)	–
Venous varices	1 (2.0)	–	–	1 (3.6)	–

SOC and the related values are provided in bold.

### Disproportional analysis

**Figure 3:**
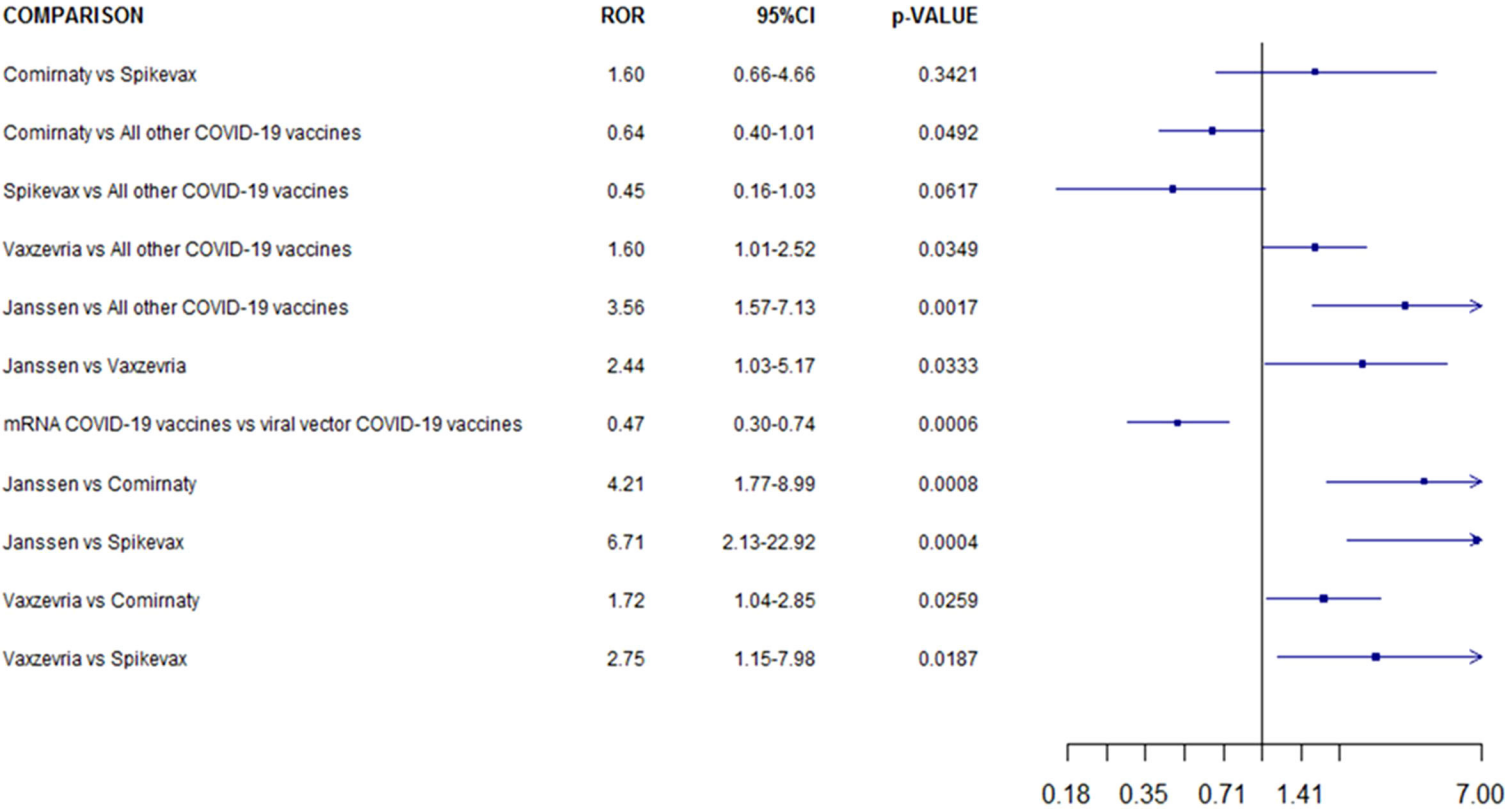
Reporting odds ratio of COVID-19 Vaccines ICSRs with capillary leak syndrome collected in the EudraVigilance spontaneous reporting system from January 1_st_ 2021 to 14_th_ January 2022 .

COVID-19 mRNA vaccines were associated to a lower CLS reporting probability compared to COVID-19 viral vector vaccines (ROR 0.5, 95% CI 0.3–0.7; p <0.001). Moreover, Ad26.COV2-S was associated with a higher reporting frequency of ICSRs with CLS when compared to BNT162b2, mRNA-1273 and ChAdOx1-SARS-COV-2 (ROR 4.21, 95% CI 1.77–8.99, p <0.001; ROR 6.71, 95% CI, 2.13–22.92, p <0.001; ROR 2.44, 95% CI 1.03–5.17; p=0.03, respectively). Both ChAdOx1-SARS-COV-2 than Ad26.COV2-S showed a higher reporting probability of CLS when compared versus other COVID-19 vaccines (ROR 1.6, 95% CI 1.01–2.52; ROR 3.56, 95% CI 1.57–7.13 respectively). No other significant statistically difference was observed (**Figure 3**).

## Discussion

Vaccines can be associated to the occurrence of AEFIs, some of which expressing a clear correlation with their action mechanisms, while other ones need further investigations and insights on the underlying mechanisms. Regardless of their mechanism or used platforms, the most commonly reported AEFIs for all COVID-19 vaccines were generally mild and transient, including injection site pain, fatigue, headache, myalgia and chills, arthralgia, pyrexia. As for all medicines, the safety monitoring of COVID-19 vaccines in the clinical practice context allows the rapid detection of rarer adverse event. Anti-COVID19 vaccines received significant media attention, inducing vaccine hesitancy among the general population, but also turning the spotlight on pharmacovigilance systems and leading a greater involvement in spontaneous reporting by citizens. Just consider that more than 40% of ChAdOx1-SARS-COV-2 -related ICSRs of our dataset were send by patients. According current pharmacovigilance legislation, patients are nowadays considered an important source of safety data (22). Although, ICSRs reported by patients could be considered maybe less reliable than those reported by a healthcare professional, we hypothesized that those still followed a CLS medical diagnosis. Given the rarity and peculiarity of the considered syndrome, it is difficult to diagnose it without a medical support, able to understand the signs and symptoms of the disease and evaluate the increase in blood parameters such as levels of hematocrit and/or hemoglobin, and hypoalbuminemia condition. The increased attention certainly raised questions about need to improve risk communication, but it also contributed to the identification of rarer adverse events, like CLS. The recent EMA warnings about CLS after viral vector vaccines have prompted our choice to investigate the occurrence of this adverse event even after immunization with mRNA COVID vaccines. According to our results, only few ICSRs describing CLS have been reported in front of billion administered doses. This could underline the rarity of this adverse reaction. On the other hand, it could also be related to the limit of underreporting characterizing spontaneous reporting system and, therefore, also our study. However, since the significant clinical relevance of CLS, this rare AEFI requires careful monitoring and investigation, as well as attention by regulatory agencies and healthcare professionals.

CLS is a rare but potentially fatal condition, whose incidence is unknown (23, 24). From our analysis emerged that CLS cases related to viral vector vaccines were more frequently unfavorable. Regarding to the gender distribution, we found an increased number of ICSR regarding the adult female patients. However, there is not a clear correspondence between the gender and the onset of this syndrome, even when it followed vaccination. In fact, the literature suggests that CLS is rarely diagnosed, most often in previously healthy, middle-aged adults without a geographical or gender preponderance (25). In September 2021, the EMA investigated possible hypothesis on underlying mechanisms of CLS following vaccination, but a precise mechanism has been not yet identified. However, EMA introduced the previous diagnosis of CLS as contraindication for vaccination with ChAdOx1-SARS-COV-2 (26). It should be emphasized that the CLS exact pathogenesis remains unclear. Based on currently knowledge, systemic CLS is a rare but serious disease, characterized by capillary dysfunction with plasma leakage from blood vessels to interstitial spaces. This induces a drop in blood pressure, which can lead to serious consequences, up to death caused by generalized edema, hypovolemic shock, ischemia-reperfusion injuries, arrhythmia or multiple organ failure. The mechanism underlying this pathology seems to be linked to a decreased adhesion of the adherent junctions and tight junctions between endothelial cells, thus resulting in the leakage of plasma. The possible hypothesized causes of the capillary loss syndrome can be diverse. One of the triggers is related to the inflammatory process. This might be the overlapping plot between CLS and COVID-19 vaccination.

**Figure 4:**
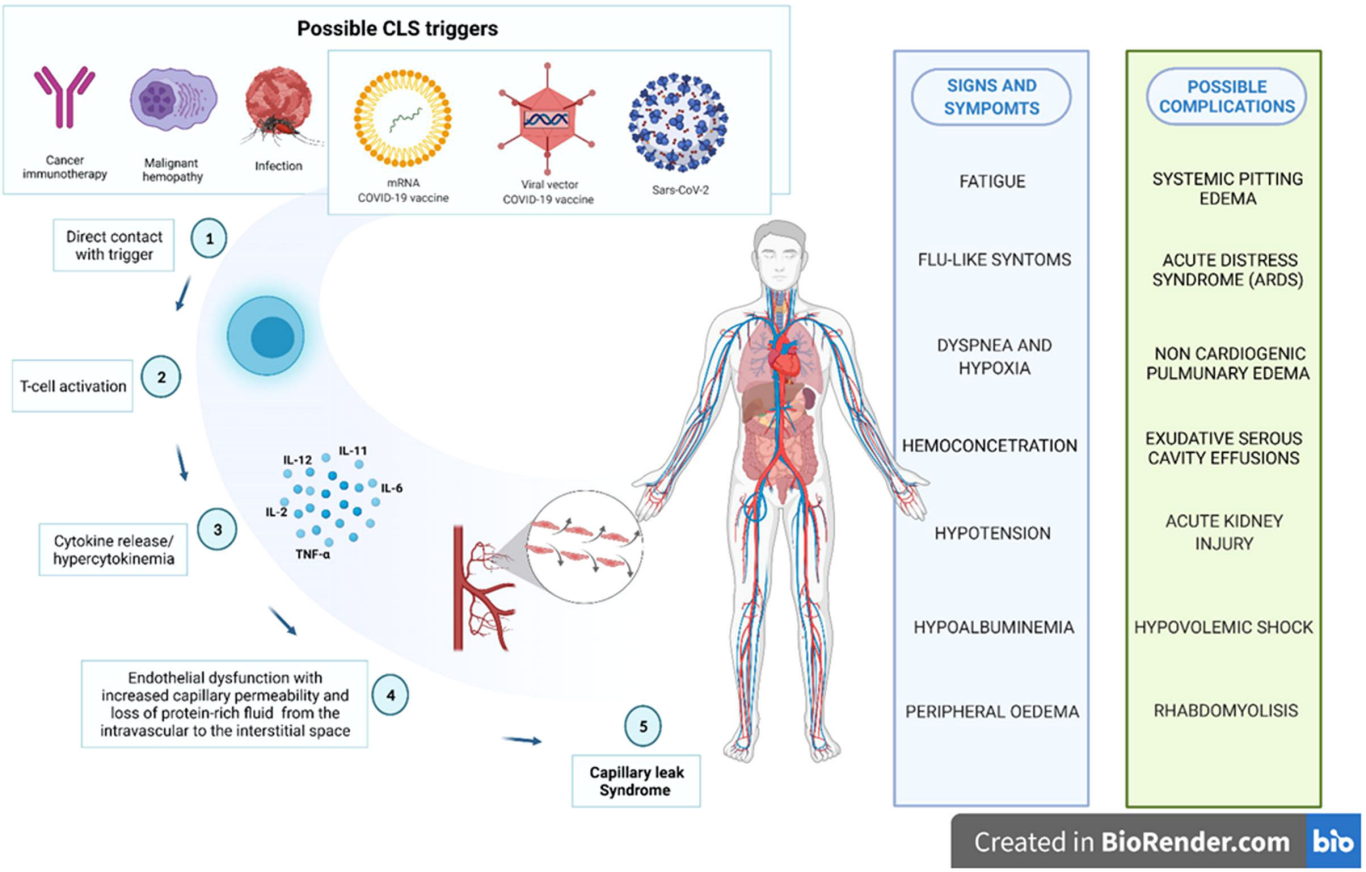
Possible biological plausibility of capillary leak syndrome induced by several triggers, including COVID-19 vaccines.

CLS can be induced either by some diseases or previous conditions or by some drugs, in particular anti-tumoral therapies (27). According to a recent systematic review focused on CLS occurred in cancer patient, in 43.6% of cases CLS was related to the cancer itself and the 51,6% of CLS cases were caused by anti-cancer agents (28). In particular, CLS has been reported as adverse effect of some monoclonal antibodies, including immunotherapy with human recombinant Interleukin-2 (IL-2), checkpoint inhibitors (anti-PD1) and rituximab (27, 29). CLS has been one of the major limit of IL-2 cancer immunotherapy, since it is a well-known adverse event of systemic administration of IL-2 at high doses (30). In our dataset, none ICSR reported anticancer agents or other concomitant drugs reasonably considered as predisposing factors or alternative causes of CLS. In addition to cancer, CLS can be caused by several syndromes including dengue infection and autoimmune diseases (**Figure 4**) (24, 27). Unfortunately, our data source does not provide us with data on the patients' previous medical histories. Recently, also COVID-19 itself has been reported to trigger first episodes and relapses of CLS, probably due to a direct toxicity of the virus SARS-CoV-2 on vascular endothelium permeability (24). In literature, several case reports and series of SARS-CoV-2 infection-induced CLS are described, including fatal relapse (31) but also favorable evolution in young patient with no significant medical history (32). In this case, the syndrome seems to be a consequence of massive secretion of pro-inflammatory cytokines in response to SARSCoV-2, resulting in a disruption of adherent junctions of endothelial cells and in a consequent increased vascular permeability (33).

Inflammatory cytokines release may have a critical role also in the biological relationship between CLS and COVID-19 vaccines. Based on currently literature knowledge, IL-2, IL-11 and tumor necrosis factor (TNF) seem to be involved in drug-induced CLS pathophysiology (24, 27). In particular, IL-2, followed by interferon (IFNγ) and TNF are implicated in endothelial damage through activation of natural killer cells. Increased IL-2 levels can induce an overproduction of nitric oxide (NO), inducing vasodilatation and systemic hypotension linked to a loss of liquids to capillary level and responsible of a cytotoxic action on endothelial cells (34). According to the immunological mechanisms, both viral vector and mRNA COVID-19 vaccines induced a strong activation of CD8+ and CD4+ T cells, as well as the production of IFNγ and IL-2. After a vitro stimulation with S protein peptides, T cell response secreting TNF and/or IL-2 (35).

Regarding the CLS management, therapeutic management include prudent intravascular expansion with crystalloids firstly and eventually albumin, preventive heparin should be administered to prevent thromboembolic events. According the cytokine-involvement hypothesis, anti-cytokine targeted therapy with infliximab, as well as steroids can improve sign and symptoms of CLS thanks the induced cytokine-downregulation (24, 36).

Finally, our results suggest a decreased CLS-reporting frequency after COVID-19 vaccination with mRNA vaccines compared to viral-vector ones. The cause of this lower frequency remains essentially unknown. Recently, also regulatory agencies have tried to identify a causal link between the mRNA vaccine mRNA-1273 and CLS occurrence. Even if there was insufficient evidence to establish a causal correlation between mRNA vaccination and the onset of new cases of CLS, it is reasonable to suppose that COVID vaccines can probably exacerbate CLS in patients with previous diagnosis, regardless the used platform vaccine (37). For this reason, the Pharmacovigilance Risk Assessment Committee (PRAC) of EMA has recommended to add a new warning for flare-ups of CLS in the product information of mRNA-1273. This PRAC recommendation was deemed necessary in order to raise awareness of the potential risk of flare-ups both healthcare professionals and patients (38). In the same way, in July 2021 EMA advised about not using Ad26.COV2-S vaccine in patients with CLS. We can’t exclude that the reporting of CLS cases following COVID-19 vaccination was influenced after EMA warnings. However, based on literature data, Regulatory Agencies alerts don’t significantly influence the reporting trends before (39). It might be interesting to verify if this was different for COVID-19 vaccines. Considering that a precise mechanism has been not yet identified, further invastigations are needed to better characterize the safety of COVID-19 vaccines.

## Strengths and limitations

Our study features intrinsic limitations of the spontaneous reporting systems. Although they provide data referred to a real-life context, allowing to a better characterization of drug safety profiles and overcoming intrinsic limits of clinical trials, spontaneous reporting systems are characterized by some limitations including the possible underreporting, low quality or lack of data. Despite these limitations, the analysis of real-life data using large databases, like Eudravigilance, allows to promptly detection of potentially safety signal. The advanced important hypotheses regarding safety drugs and vaccines require further *ad-hoc* confirmatory studies, based on different study design. Therefore, although pharmacovigilance studies are useful for the identification of new safety signals, further investigation are necessary in order to verify the causal association between a drug/vaccine and the risk of a specific adverse events. Overall, pharmacovigilance database analysis contributed to regulatory actions aimed to guarantee safer use of medicines, how it was highlighted during the pandemic. Disproportionality analysis of pharmacovigilance databases is a validated, quick and inexpensive method useful to confirm a potential association between a drug and an ADR, or identify rare and/or new ADR. Disproportionality analysis has been also used to validate some pharmacological hypotheses regarding the mechanism of occurrence of ADRs (40). However, regarding this latter aspect, it should not be underestimated or forgotten that the cases reported by both healthcare professionals and patients are subject to validation by local pharmacovigilance managers.

## Conclusions

During the study period, we retrieved 84 ICRs described CLS and mainly referred to ChAdOx1-SARS-COV-2, followed by BNT162b2, Ad26.COV2-S and mRNA-1273 . This limited number of ICSRs collected in EV and describing CLS in front of billion administered doses underlines the rarity of this adverse reaction. However, it is likely that many cases of capillary leak are unrecognized (24). The low number of ICSRs with CLS as AEFI and COVID-19 mRNA or viral vector vaccines as a suspected drug should be contextualized with the rarity of the disease. On the other hand, its significant clinical relevance requires careful monitoring and investigation, as well as attention by regulatory agencies and healthcare professionals. It may be very important alert clinicians to the possibility of CLS-like events after COVID-19 vaccination with the aim of minimize the risk, especially in the population with a medical history of this condition. According with other recent studies, our results highlighted that both viral vector and mRNA vaccines could be potentially associated to systemic events such as capillary leak syndrome. Our analysis, even if subject to the limitations of spontaneous reporting systems, suggests a small but statistically significant safety concern for CLS following receipt of COVID-19 viral vector vaccines, in particular Ad26.COV2-S. Cytokine-release following T-cell activation could be involved in the occurrence of these AEFI, but a precise mechanism has been not yet identified. COVID-19 vaccines remain attentive as possible triggers of CLS. Healthcare professionals as well as patients/citizens should be aware of the signs and symptoms of CLS and of a possible risk of flare-ups in people with a history of CLS, which require particular attention. Further studies are needed to identify the causal relationship and the mechanisms underlying the onset of CLS after COVID-19 vaccination, nowadays still unclear. Even if a precise mechanism has been not yet identified, COVID-19 vaccines are actually attentive as possible triggers of CLS (**Figure 4**). Constant pharmacovigilance activities together with all other research networks can lead to better characterization of this safety issue of COVID-19 vaccines.

## Data availability statement

The datasets presented in this study can be found in online repositories. The names of the repository/repositories and accession number(s) can be found below: www.adrreports.eu.

## Ethics statement

Ethical review and approval was not required for the study on human participants in accordance with the local legislation and institutional requirements. Written informed consent for participation was not required for this study in accordance with the national legislation and the institutional requirements.

## Author contributions

Concept and design, FR and AC. Acquisition, analysis, or interpretation of data, RR, NB, RN, and PB. Drafting of the manuscript, RR, NB, and RN. Critical revision of the manuscript for important intellectual content, AM, CR, and LS. Statistical analysis, RR, NB, RN, and AM. Supervision, FR and AC. All authors contributed to the article and approved the submitted version.

## Conflict of interest

The authors declare that the research was conducted in the absence of any commercial or financial relationships that could be construed as a potential conflict of interest.

## Publisher’s note

All claims expressed in this article are solely those of the authors and do not necessarily represent those of their affiliated organizations, or those of the publisher, the editors and the reviewers. Any product that may be evaluated in this article, or claim that may be made by its manufacturer, is not guaranteed or endorsed by the publisher.

## References

[1] Chang L Yan Y Wang L. Coronavirus disease 2019: Coronaviruses and blood safety. Transfus Med Rev (2020) 34(2):75–80. doi: 10.1016/J.TMRV.2020.02.003 PMC7135848 32107119

[2] Alimohamadi Y Sepandi M Taghdir M Hosamirudsari H. Determine the most common clinical symptoms in COVID-19 patients: A systematic review and meta-analysis. J Prev Med Hyg (2020) 61(3):E304–12. doi: 10.15167/2421-4248/jpmh2020.61.3.1530 PMC7595075 33150219

[3] Scavone C Mascolo A Rafaniello C Sportiello L Trama U Zoccoli A. Therapeutic strategies to fight COVID-19: Which is the status artis? Br J Pharmacol (2021). doi: 10.1111/BPH.15452 PMC8239658 33960398

[4] Sultana J Mazzaglia G Luxi N Cancellieri A Capuano A Ferrajolo C. Potential effects of vaccinations on the prevention of COVID-19: Rationale, clinical evidence, risks, and public health considerations. Expert Rev Vaccines (2020) 19(10):919–36. doi: 10.1080/14760584.2020.1825951 32940090

[5] Scavone C Brusco S Bertini M Sportiello L Rafaniello C Zoccoli A. Current pharmacological treatments for COVID-19: What’s next? Br J Pharmacol (2020) 177(21):4813–24. doi: 10.1111/BPH.15072 PMC7264618 32329520

[6] Kaur SP Gupta V. COVID-19 vaccine: A comprehensive status report. Virus Research (2020) 15(288):198114. doi: 10.1016/j.virusres.2020.198114 PMC7423510 32800805

[7] Wagner R Hildt E Grabski E Sun Y Meyer H Lommel A. Accelerated development of covid-19 vaccines: Technology platforms, benefits, and associated risks. Vaccines (Basel) (2021) 9(7):1–11. doi: 10.3390/vaccines9070747 PMC8310218 34358163

[8] Lamb YN. BNT162b2 mRNA COVID-19 vaccine: First approval. Drugs (2021) 81(4):495–501. doi: 10.1007/S40265-021-01480-7 PMC7938284 33683637

[9] European Medicines Agency. COVID-19 vaccines | European medicines agency . Available at: https://www.ema.europa.eu/en/human-regulatory/overview/public-health-threats/coronavirus-disease-covid-19/treatments-vaccines/covid-19-vaccines (Accessed March 14, 2022).

[10] Rafaniello C Ferrajolo C Sullo MG Gaio M Zinzi A Scavone C. Cardiac events potentially associated to remdesivir: An analysis from the european spontaneous adverse event reporting system. Pharmaceuticals (2021) 14(7):611. doi: 10.3390/PH14070611/S1 34202350 PMC8308754

[11] Sultana J Mazzaglia G Luxi N Cancellieri A Capuano A Ferrajolo C. Potential effects of vaccinations on the prevention of COVID-19: Rationale, clinical evidence, risks, and public health considerations. Expert Rev Vaccines (2020) 19(10):919–36. doi: 10.1080/14760584.2020.1825951 32940090

[12] Matheny M Maleque N Channell N Eisch AR Auld SC Banerji A. Severe exacerbations of systemic capillary leak syndrome after covid-19 vaccination: A case series. Ann Internal Med (2021) 174(10):1476–8. doi: 10.7326/L21-0250 PMC8252024 34125573

[13] Choi GJ Baek SH Kim J Kim JH Kwon GY Kim DK. Fatal systemic capillary leak syndrome after SARS-CoV-2Vaccination in patient with multiple myeloma. Emerg Infect Dis (2021) 27(11):2973–75. doi: 10.3201/EID2711.211723 PMC8544977 34459725

[14] Buj M Morales-Varas G Pedrosa-Guerrero A Alonso-Ciria E. Systemic capillary leak syndrome after SARS-CoV-2 infection and after COVID-19 vaccination: A scoping review in relation to a clinical case. Rev Clin Esp (2022) 222(6):374–376. doi: 10.1016/j.rceng.2021.11.005 PMC8882416 35256311

[15] Robichaud J Côté C Côté F. Systemic capillary leak syndrome after ChAdOx1 nCOV-19 (Oxford-AstraZeneca) vaccination. CMAJ (2021) 193(34):E1341–4. doi: 10.1503/CMAJ.211212 PMC8432311 34362727

[16] Pineton de Chambrun M Luyt CE Beloncle F Gousseff M Mauhin W Argaud L. The clinical picture of severe systemic capillary-leak syndrome episodes requiring ICU admission. Crit Care Med (2017) 45(7):1216–23. doi: 10.1097/CCM.0000000000002496 28622216

[17] European Medicines Agency. COVID-19 vaccine safety update VAXZEVRIA AstraZeneca AB. (2021). Available at : https://www.ema.europa.eu/en/documents/covid-19-vaccine-safety-update/covid-19-vaccine-safety-update-vaxzevria-previously-covid-19-vaccine-astrazeneca-29-march-2021_en.pdf. (Accessed July,14 2022).

[18] European Medicines Agency. EMA advises against use of COVID-19 vaccine janssen in people with history of capillary leak syndrome | European medicines agency. Available at: https://www.ema.europa.eu/en/news/ema-advises-against-use-covid-19-vaccine-janssen-people-history-capillary-leak-syndrome (Accessed March 14, 2022).

[19] Buj M Morales-Varas G Pedrosa-Guerrero A Alonso-Ciria E. Systemic capillary leak syndrome after SARS-CoV-2 infection and after COVID-19 vaccination: A scoping review in relation to a clinical case. Rev Clínica Española (English Edition) (2022) 222(6):374–6. doi: 10.1016/J.RCENG.2021.11.005 PMC8882416 35256311

[20] Inoue M Yasue Y Kobayashi Y Sugiyama Y. Systemic capillary leak syndrome (SCLS) after receiving BNT162b2 mRNA COVID-19 (Pfizer-BioNTech) vaccine. BMJ Case Rep (2022) 15(3):e248927. doi: 10.1136/BCR-2022-248927 PMC8928276 35292552

[21] Bihan K Lebrun-Vignes B Funck-Brentano C Salem JE. Uses of pharmacovigilance databases: An overview. Therapie (2020) 75(6):591–598. doi: 10.1016/J.THERAP.2020.02.022 32169289

[22] Sienkiewicz K Burzyńska M Rydlewska-Liszkowska I Sienkiewicz J Gaszyńska E. The importance of direct patient reporting of adverse drug reactions in the safety monitoring process. Int J Environ Res Public Health (2022) 19(1):413. doi: 10.3390/IJERPH19010413 PMC8745009 35010673

[23] Kapoor P Greipp PT Schaefer EW. Idiopathic systemic capillary leak syndrome (Clarkson’s disease): The Mayo clinic experience. Mayo Clinic Proc (2010) 85(10):905–12. doi: 10.4065/MCP.2010.0159 PMC2947962 20634497

[24] Siddall E Khatri M Radhakrishnan J. Capillary leak syndrome: etiologies, pathophysiology, and management. Kidney Int (2017) 92(1):37–46. doi: 10.1016/J.KINT.2016.11.029 28318633

[25] Druey KM Greipp PR. Narrative review: Clarkson disease-systemic capillary leak syndrome. Ann Intern Med (2010) 153(2):90. doi: 10.1059/0003-4819-153-2-201007200-00005 PMC3017349 20643990

[26] European Medicines Agency. COVID-19 vaccine safety update VAXZEVRIA AstraZeneca AB. (2021). Available at: https://www.ema.europa.eu/en/documents/covid-19-vaccine-safety-update/covid-19-vaccine-safety-update-vaxzevria-previously-covid-19-vaccine-astrazeneca-8-september-2021_en.pdf (Accessed March 14, 2022).

[27] Bichon A Bourenne J Gainnier M Carvelli J. Capillary leak syndrome: State of the art in 2021. Rev Med Interne. (2021) 42(11):789–96. doi: 10.1016/J.REVMED.2021.05.012 34099313

[28] Shin JI Lee KH Lee IR Oh JH Kim DW Shin JW. Systemic capillary leak syndrome (Clarkson syndrome) in cancer patients: A systematic review. J Clin Med (2018) 7(11):418. doi: 10.3390/JCM7110418 PMC6262589 30404164

[29] Jeong GH Lee KH Lee IR Oh JH Kim DW Shin JW. Incidence of capillary leak syndrome as an adverse effect of drugs in cancer patients: A systematic review and meta-analysis. J Clin Med (2019) 8(2):143. doi: 10.3390/JCM8020143 PMC6406478 30691103

[30] Raggi G Roldan N Micallef V Rapet A De Maddalena L Imler T. Interleukin-2 – induced vascular leak syndrome: clinically relevant *in vitro* recapitulation with a patient-derived lung-on-chip. Eur Respir J (2020) 56(suppl 64):4326. doi: 10.1183/13993003.CONGRESS-2020.4326

[31] Pineton de Chambrun M Cohen-Aubart F Donker DW Cariou PL Luyt CE Combes A. SARS-CoV-2 induces acute and refractory relapse of systemic capillary leak syndrome (Clarkson’s disease). Am J Med (2020) 133(11):e663–4. doi: 10.1016/J.AMJMED.2020.03.057 PMC7217792 32405072

[32] Lacout C Rogez J Orvain C Nicot C Rony L Julien H. A new diagnosis of systemic capillary leak syndrome in a patient with COVID-19. Rheumatol (Oxford). (2021) 60(1):E19–20. doi: 10.1093/RHEUMATOLOGY/KEAA606 PMC7543632 32940700

[33] Mehta P McAuley DF Brown M Sanchez E Tattersall RS Manson JJ. COVID-19: consider cytokine storm syndromes and immunosuppression. Lancet (2020) 395(10229):1033–4. doi: 10.1016/S0140-6736(20)30628-0 PMC7270045 32192578

[34] Orucevic A Lala PK. Role of nitric oxide in IL-2 therapy-induced capillary leak syndrome. Cancer Metastasis Rev 1998;17(1):127–42. doi: 10.1023/a:1005969024182. 9544428

[35] Sadarangani M Marchant A Kollmann TR. Immunological mechanisms of vaccine-induced protection against COVID-19 in humans. Nat Rev Immunol (2021) 21(8):475–84:8. doi: 10.1038/s41577-021-00578-z PMC8246128 34211186

[36] Bichon A Bourenne J Gainnier M Carvelli J. Capillary leak syndrome: State of the art in 2021. La Rev Médecine Interne (2021) 42(11):789–96. doi: 10.1016/J.REVMED.2021.05.012 34099313

[37] Roncati L Gianotti G Ambrogi E Attolini G. Capillary leak syndrome in COVID-19 and post COVID-19 vaccines. Eur J Gynaecol Oncol (2021) 42(5):829–31. doi: 10.31083/J.EJGO4205126/0392-2936-42-5-829/FIG1.JPG

[38] European Medicines Agency. Meeting highlights from the pharmacovigilance risk assessment committee (PRAC) 7-10 march 2022 | European medicines agency . Available at: https://www.ema.europa.eu/en/news/meeting-highlights-pharmacovigilance-risk-assessment-committee-prac-7-10-march-2022 (Accessed March 14, 2022).

[39] Hoffman KB Demakas AR Dimbil M Tatonetti NP Erdman CB. Stimulated reporting: The impact of US food and drug administration-issued alerts on the adverse event reporting system (FAERS). Drug Saf (2014) 37(11):971–80. doi: 10.1007/s40264-014-0225-0 PMC4206770 25255848

[40] Montastruc JL Sommet A Bagheri H Lapeyre-Mestre M. Benefits and strengths of the disproportionality analysis for identification of adverse drug reactions in a pharmacovigilance database. Br J Clin Pharmacol (2011) 72(6):905–8. doi: 10.1111/J.1365-2125.2011.04037.X PMC3244636 21658092

